# Discovery of nitric oxide and cyclic GMP in cell signaling and their role in drug development

**DOI:** 10.1186/1750-1326-7-S1-L1

**Published:** 2012-02-07

**Authors:** Ferid Murad

**Affiliations:** 1 George Washington University, Washington, D.C., USA

## 

The role of nitric oxide in cellular signaling in the past three decades has become one of the most rapidly growing areas in biology. Nitric oxide is a gas and a free radical with an unshared electron that can regulate an ever-growing list of biological processes. Nitric oxide is formed from L-arginine by a family of enzymes called nitric oxide synthases. These enzymes have a complex requirement for a number of cofactors and regulators including NADPH, tetrahydrobioterin, flavins, calmodulin and heme. The enzymes are present in most cells and tissues. In many instances, nitric oxide mediates its biological effects by activating the soluble isoform of guanylyl cyclase and increasing cyclic GMP synthesis from GTP. Cyclic GMP, in turn, can activate cyclic GMP-dependent protein kinase (PKG) and can cause smooth muscles and blood vessels to relax, decrease platelet aggregation, alter neuron function, etc. These effects can decrease blood pressure, increase blood flow to tissues, alter memory and behavior, decrease blood clotting, etc. The list of effects of nitric oxide that are independent of cyclic GMP formation is also growing at a rapid rate. For example, nitric oxide can interact with transition metals such as iron, thiol groups, other free radicals, oxygen, superoxide anion, unsaturated fatty acids, and other molecules. Some of these reactions result in the oxidation of nitric oxide to nitrite and nitrate to terminate the effect, while other reactions can lead to altered protein structure function and/or catalytic capacity. These effects probably regulate bacterial infections, inflammation of tissues, tumor growth, and other disorders. These diverse effects of nitric oxide that are cyclic GMP dependent or independent can alter and regulate numerous important physiological events in cell regulation and function. Nitric oxide can function as an intracellular messenger, an antacoid, a paracrine substance, a neurotransmitter, or as a hormone that can be carried to distant sites for effects. Thus, it is a unique molecule with an array of signaling functions. However, with any messenger molecule, there can be too little or too much of the substance, resulting in pathological events. Some of the methods to regulate either nitric oxide formation metabolism, or function have been in clinical use for more than a century, as with the use of organic nitrates and nitroglycerin in angina pectoris that was initiated in the 1870s. Inhalation of low concentrations of nitric oxide can be beneficial in premature infants with pulmonary hypertension and increase survival rates. Ongoing clinical trials with nitric oxide synthase inhibitors and nitric oxide scavengers are examining the effects of these agents in septic shock, hypotension with dialysis, inflammatory disorders, cancer therapy, etc. Recognition of additional molecular targets in the areas of nitric oxide and cyclic GMP research will continue to promote drug discovery and development programs in the field. Current and future research will undoubtedly expand the clinician’s therapeutic armamentarium to manage a number of important diseases by perturbing nitric oxide formation and metabolism. Such promise and expectations have obviously fueled the interests in nitric oxide research for a growing list of potential therapeutic applications. There have been and will continue to be many opportunities from nitric oxide and cyclic GMP march to develop novel and important therapeutic agents. There are presently more than 80,000 publications in the area of nitric oxide research. The lecture will discuss our discovery of the first biological effects of nitric oxide and how the field has evolved since our original reports in 1977. The possible utility of this signaling pathway to facilitate novel drug development and the creation of numerous projects in the Pharmaceutical and biotechnology industrials will also be discussed.

